# Aberrant Right Hepatic Duct Draining into the Cystic Duct: Clinical Outcomes and Management

**DOI:** 10.1155/2011/458915

**Published:** 2011-04-07

**Authors:** Aijaz A. Sofi, Osama H. Alaradi, Marwan Abouljoud, Ali T. Nawras

**Affiliations:** ^1^Division of Gastroenterology, Department of Medicine, University of Toledo Medical Center, 3000 Arlington Avenue, Toledo, OH 43614, USA; ^2^Department of Gastroenterology, Henry Ford Health System, Detroit, MI 48202, USA; ^3^Transplant Institute, Henry Ford Health System, Detroit, MI 48202, USA

## Abstract

*Background*. Aberrant right hepatic duct (ARHD) draining into cystic duct (CD) is relatively rare but clinically important because of its susceptibility to injuries during cholecystectomy. These injuries are often-times missed or diagnosed late and as a result can develop serious complications. *Methods*. Four consecutive patients diagnosed with ARHD draining into CD were identified for inclusion. *Results*. The mean age of patients was 42.5 years. The diagnosis in one of the patient was incidental during a routine endoscopic retrograde cholangiopancreatography (ERCP). Other three patients were diagnosed post-cholecystectomy- one presented with suspected intra-operative biliary injury, one with persistent bile leak and another with recurrent cholangitis. Inadequate filling of the segment of liver on ERCP with dilation of intrahepatic ducts in the corresponding segment on imaging was present in two patients with complete obstruction of ARHD which was managed surgically. In another patient, the partially obstructed ARHD was managed by endoscopic therapy. *Conclusion*. ARHD draining into the CD can have varied clinical manifestations. In appropriate clinical settings, it should be suspected in patients with persistence of bile leak early after cholecystectomy, segmental dilation of intrahepatic-bile ducts on imaging and paucity of intrahepatic filling in a segment of liver on ERCP.

## 1. Introduction

Bile duct injury is an uncommon complication following cholecystectomy. With the increasing use of laparoscopic cholecystectomy (LC), there has been an associated increase in the incidence of bile duct injuries [1]. Early studies reported a decline in the injury rate which was attributed to the phenomenon referred to as “learning curve” [2]. However, several subsequent studies suggest that the decline was not sustained [3, 4]. Anatomic variations of biliary tree was identified as one of the risk factor for bile duct injury following LC [5]. 

 Therefore, the knowledge of various biliary anomalies and their early identification may further assist in decreasing the rate of biliary tract injuries. Here we present a case-series on various presentations and management of a rare anomaly of biliary tree.

**Case 1**

**Figure 1:**
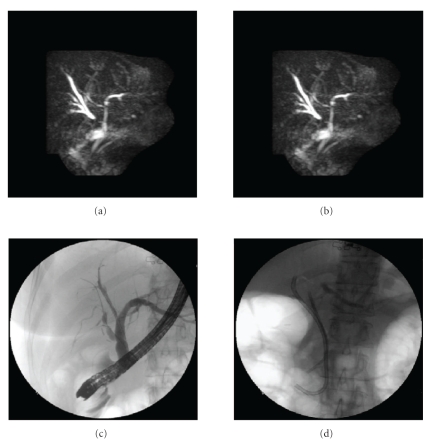
(a) Endoscopic retrograde cholangiopancreatography (ERCP) showing aberrant right hepatic duct filled through cystic duct stump. (b) Magnetic resonance cholangiopancreatography demonstrating dilated intrahepatic ducts in the right lobe of liver. (c) the guidewire placed across the stricture in the aberrant right aberrant bile duct for balloon dilation. (d) Two biliary stents placed-one through the cystic stump into the aberrant right hepatic duct and the other through the common hepatic duct into the right hepatic biliary system.

A 55-year-old woman presented 3 days postcholecystectomy (LC) with upper abdominal pain. HIDA scan revealed a bile leak. ERCP with minimal contrast injection revealed a leak from cystic stump; therefore a 10 F biliary stent was placed. Six weeks later a repeat ERCP revealed no leak from the cystic duct stump. However, large branch of the right intrahepatic duct was filled through the cystic duct stump (Figure 1(a)). Minimally abnormal liver function tests (LFTs) were noted on 4 months followup. Magnetic resonance cholangiopancreatography (MRCP) showed some dilated branches of right intrahepatic duct (Figure 1(b)), warranting a repeat ERCP. At this time a stricture was found at the site where the cystic duct stump was communicating with the branch of the right intrahepatic duct. Guide wire was passed into that branch through the cystic duct stump in addition to another guide wire up to the right hepatic duct through the common hepatic duct (CHD) (Figure 1(c)). The stricture was then dilated with a balloon. Two biliary stents were placed, one through the cystic stump (10F × 12 cm) and another through the CHD into the right hepatic biliary system (7F × 7 cm) to maintain the biliary drainage (Figure 1(d)). The procedure was repeated twice within 2 months interval. Stents were removed after 4 months and LFTs were normal 2 months after stent removal. No long-term followup is available.

**Case 2**

**Figure 2:**
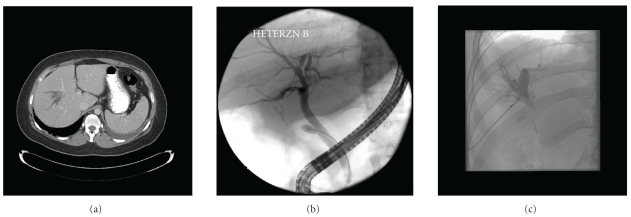
(a) Computed tomography (CT)-scan showing dilated intrahepatic branches in right lobe of liver. (b) Endoscopic retrograde cholangiopancreatography (ERCP) showing paucity of intrahepatic filling in right lobe of liver in an otherwise normal looking ERCP. (c) Percutaneous transhepatic cholangiopancreatography (PTC) showing dilation of intrahepatic branches in the liver segment drained by the aberrant branch isolated by surgical clips.

A 38-year-old Caucasian woman was referred to our hospital for recurrent episodes of gram-negative bacteremia. On presentation she was asymptomatic. Her past surgical history was significant for complicated cholecystectomy requiring conversion from laparoscopic to open technique six years earlier when she was 28 weeks pregnant. The surgical report described normal common and cystic ducts and described bile staining within the hilum where a drain was placed. After a few days drainage stopped and drain was removed. She was found to have minimally abnormal LFT's during a routine blood workup, a year after the surgery. Ultrasound at that time demonstrated dilated biliary tree in a segment of the right lobe of the liver and an ERCP revealed a questionable cystic dilation of distal common bile duct (CBD), for which sphincterotomy was performed. She had recurrent episodes of fever in the subsequent years and blood cultures persistently grew Klebsiella species. Computerized tomography (CT) scan (Figure 2(a)) and MRCP performed during one of these episodes had shown dilated intrahepatic ducts in a segment of right lobe of liver, most likely the posterior sector. ERCP repeated 4 years later at another center was reported normal. However, ERCP performed at our center revealed normal CBD and cystic duct stump with inadequate filling of intrahepatic ducts in a segment of right hepatic lobe, which corresponded to the dilated “blind” duct segment seen on MRCP (Figure 2(b)). Percutaneous transhepatic cholangiography (PTC) was performed to further evaluate the dilated branches of right intrahepatic bile duct (Figure 2(c)) as seen on MRCP and CT scan. Obstruction appeared to be at the level of the surgical clips which had been applied to cystic duct at the time of cholecystectomy. This obstruction could not be traversed with a catheter or guide-wire after several attempts. During the operative procedure, a fibrosed ductal structure with clips was mobilized in the hilum and tracked to the cystic duct stump. Proximally, there was extensive sclerosis. Liver bed was partially dissected and the intrahepatic portion of the right posterior sector was isolated. A Roux-en-Y hepatico-jejunostomy to the right aberrant hepatic duct was performed without difficulty. Based on the aforementioned findings with an intact right anterior sector hepatic duct and common hepatic duct, presence of one set of clips along the common hepatic duct (negating presence of an additional stump for the right posterior sectoral duct) combined with the intraoperative findings, the patient was diagnosed with an aberrant right hepatic duct draining into cystic duct that was clipped/injured during cholecystectomy.

**Case 3**

**Figure 3:**
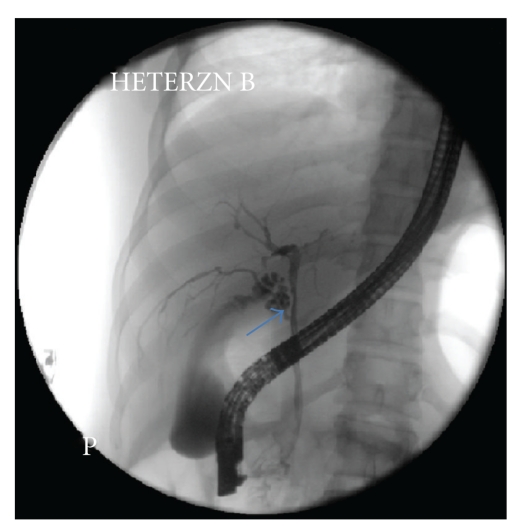
Endoscopic retrograde cholangiopancreatography (ERCP) demonstrating aberrant right hepatic duct draining a segment of right lobe of liver and emptying into cystic duct (*arrow*).

A 27-year-old healthy young woman with history of generalized fatigue presented for further evaluation of abnormal liver biochemistries. Her alkaline phosphatase levels had nearly increased 3 times the upper limits of normal. The most recent LFT revealed elevation of aspartate aminotransferases (AST) (104 U/L) and alanine aminotransferases (ALT) (234 U/L), which were reported as normal two years ago. An abdominal ultrasound showed no focal lesions. MRCP was performed which did not reveal any abnormal findings. Clinical examination was normal. In view of strong clinical suspicion of sclerosing cholangitis, ERCP was performed which revealed diffuse multifocal strictures with beading of intrahepatic ducts bilaterally, suspicious for primary sclerosing cholangitis. An aberrant branch of right hepatic duct draining into CBD with cystic duct originating from the aberrant branch was noted on ERCP (Figure 3).

**Case 4**

**Figure 4:**
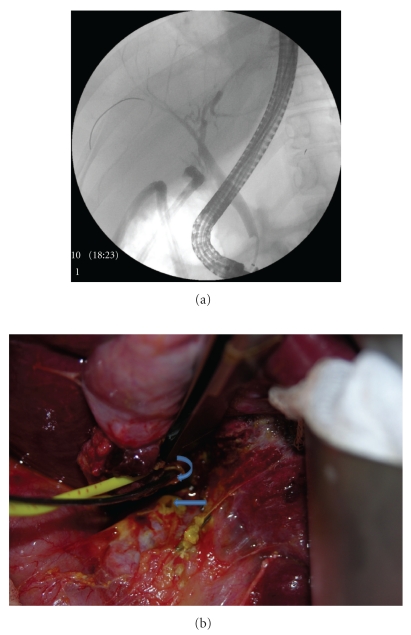
(a) Endoscopic retrograde cholangiopancreatography (ERCP) showing staples close to common bile duct with inadequate filling of intrahepatic ducts in right lobe of liver. (b) Operative photograph-probe in the torn aberrant duct (*curved arrow*) and ostium on the surface of cystic duct stump (*line arrow*).

A 50-year-old woman presented with symptomatic cholelithiasis. Elective LC was attempted at an outside facility. Intraoperative cholangiogram (IOC) was interpreted as-right intrahepatic ducts filling without opacification of mid-CBD and CHD. Surgery was aborted in view of suspicion of bile duct injury. Two JP (Jackson-Pratt) drains were placed and she was referred to our center. ERCP performed at our center revealed cystic duct remnant leak and staples were noted close to CBD. There was inadequate filling of intrahepatic ducts in a segment of right hepatic lobe (Figure 4(a)). Sphincterotomy was performed and biliary stents were placed in right hepatic and left hepatic ducts. Patient was again taken for surgery for suspected aberrant right hepatic duct, which was identified during surgery and was found to be cauterized. In addition, an ostium on the surface of cystic duct was identified (Figure 4(b)). A Roux-en-Y hepatico-jejunostomy to the right aberrant hepatic duct was performed. Biliary stents were removed 2 months after the surgery. She was asymptomatic 4 months after the surgery and her LFT's remained normal.

## 2. Discussion

Aberrant right hepatic duct (ARHD) is branch providing biliary drainage to variable portion of right hepatic lobe and drains directly into the extrahepatic biliary tree. ARHD is a common bile tract anomaly with the incidence of 4.6%–8.4% and it frequently drains into common hepatic duct, CBD or even left hepatic duct [6, 7]. However, the anomalous drainage of aberrant right hepatic duct into cystic duct is relatively rare but crucial in view of its susceptibility to injury during cholecystectomy. In a study by Peunte et al. this abnormality was seen in one of 4264 patients who had operative cholangiograms [6]. Another study by Kullman et al. found this anomaly in 2.9% of 513 patients who underwent routine IOC [7]. The operative injury to these ducts can result in bile leak if torn or obstruction of bile tract of the corresponding hepatic segments leading to segmental biliary cirrhosis. Furthermore, obstruction of biliary tree usually results in recurrent episodes of cholangitis in these patients [8]. 

IOC during LC has been utilized to identify anatomic variants of biliary tract, even though it's routine use is debatable [9]. While some of the studies failed to show any benefit of IOC in preventing bile duct injury in these patients [10], few others have shown it to be useful [7, 11]. However, human error is an important factor of failure to identify bile-duct injury or aberrant anatomy during IOC [12]. This is evident in our fourth case, where, filling of intrahepatic ducts in right lobe of liver was noted on IOC but was not interpreted correctly. Therefore, these patients usually present with postcholecystectomy bile duct injuries.

Persistent bile leak may be an initial presentation of injury to the ARHD during cholecystectomy [13]. These patients usually present with abdominal pain or fever if biloma becomes infected. Abdominal ultrasound or CT-scan may reveal perihepatic fluid collection. Such patients usually undergo ERCP and placement of biliary stents. Although ERCP is helpful in delineating biliary anomalies, it usually fails to provide diagnosis after cholecystectomy when aberrant ducts have been severed [8] which is also evident in our series. This is because the segmental bile ducts usually lack intercommunication [14]. In these cases, particular attention should be paid to paucity of intrahepatic biliary filling in a segment of liver. This would be a useful sign to suspect aberrant bile duct injury in these cases as in our second and fourth patient. Sometimes, a cholangiogram obtained through the drain placed in biloma has been shown to delineate a torn aberrant bile duct [13, 15].

Less frequently, patients with ARHD injury can present in the postoperative period weeks to months later, with cholestatic pattern of abnormality on LFT's, if the aberrant duct is blocked. In addition, these patients can also present with episodes of cholangitis in the obstructed segment of biliary tree as was the case with our second case. A high index of suspicion is required to look for these complications. Segmental dilation of intrahepatic ducts may be revealed on a CT-scan. These patients will require cholangiography (PTC or ERCP) to define the anatomy of biliary tree. Scantiness of intrahepatic filling on ERCP with dilation of intrahepatic branches in the same sector on radiologic imaging should be a useful clue to the diagnosis of obstruction of aberrant branch of bile duct. PTC can be performed when intrahepatic ducts are dilated; sometimes both the procedures may be required to obtain the diagnosis [16] as was the case in our second patient. Recently MRCP has been shown to be effective in diagnosing postoperative bile tract injuries [17, 18]. However, its efficacy in diagnosing aberrant bile duct injury is limited. In our series MRCP could only show dilated intrahepatic ducts in right side of liver in our first and second patient, which was already documented on CT-scan earlier, but failed to show aberrant right hepatic duct and similar observation have been reported earlier [19]. Therefore, MRCP may not be valuable in suspected aberrant bile-duct injury as it may not add any significant information to the findings of ultrasound or CT-scan.

Usually, the initial management of patients presenting with a bile leak after LC is decompression of biliary tree by placing a stent with or without sphincterotomy or placement of naso-biliary drain or percutaneous drain [20]. If an aberrant bile-duct is partially blocked, as in our first case, endoscopic treatment should be attempted to reopen the duct. However, these patients will need longer followup for potential subsequent complications particularly ductal stenosis. Endotherapy for obstructed aberrant bile ducts has been reported and the results were encouraging [21]. Surgical therapy may be required for completely obstructed aberrant ducts as in our fourth patient.

The diagnosis of aberrant right hepatic duct draining into cystic duct can be incidental during ERCP or MRCP if performed before surgery, as in our third patient. In these situations, the knowledge of aberrant anatomy of biliary track would be important for a surgeon to avoid any inadvertent complications if they undergo cholecystectomy or any other biliary surgery. In addition, the safe approach to avoid injury to aberrant bile-ducts during cholecystectomy is adhering to the gallbladder itself, identifying the triangle of Calot and using the “critical view of safety (CVS)”, as described by Strasberg, before dividing the cystic structures. CVS involves 3 steps: (i) clearing triangle of Callot of fat and fibrous tissue, (ii) separation of lowest part of GB from liver bed, (iii) only 2 structures (cystic artery and cystic duct) should be seen entering GB [22]. 

 In summary, we present a varied clinical presentation and management of a relatively rare but clinically significant anatomic variation of bile duct anatomy. Aberrant bile-duct injury is often missed as subtle signs of injury remain unrecognized both by surgeons as well as by gastroenterologists. Aberrant bile duct injury after cholecystectomy should be strongly suspected in following situations: (i) paucity of intrahepatic filling in a segment of liver on ERCP; (ii) abnormal LFTs with segmental dilation of intrahepatic-bile ducts on abdominal CT-scan or ultrasound.
